# Improving awareness and knowledge about dementia risk reduction in the general population: a Dutch public awareness campaign

**DOI:** 10.1002/alz70860_106649

**Published:** 2025-12-23

**Authors:** Niels Janssen, Dominique Paauw, Lieke Bakker, Irene Heger, Kay Deckers, Sebastian Köhler

**Affiliations:** ^1^ Department of Psychiatry and Neuropsychology, Alzheimer Centrum Limburg, Mental Health and Neuroscience Research Institute (MHeNs), Maastricht University, Maastricht, Netherlands

## Abstract

**Background:**

Around 45% of dementia cases is likely attributable to modifiable risk factors, such as hypertension and high cholesterol. It is therefore important to increase awareness on the potential of dementia risk reduction on a population level. Herein, people with low socioeconomic position or migration background are often underrepresented. We assessed levels of awareness for dementia risk reduction in the general population and underrepresented groups.

**Method:**

Online surveys and street interviews were conducted to test awareness about dementia risk being modifiable and knowledge regarding individual risk factors in the regions of South Limburg (*n* = 1619) and Groningen (*n* = 505), the Netherlands. In addition, two local districts characterized by low‐socioeconomic position (*n* = 120) or migration background (*n* = 330) were assessed. This was part of a baseline assessment within the Netherlands Dementia Prevention Initiative (NDPI) assessment, in which a newly designed awareness campaign will run from February 2025 for a total duration of 12 months in these regions and districts. The local campaigns will be implemented using a participatory, bottom‐up approach. Various activities are planned, including the use of mass media and public events.

**Result:**

Initial results of measurements before the start of the campaign show that most participants rated their own knowledge about dementia as ‘fair’ (47% and 52%). Between 49% and 71% of all respondents are aware of the potential of risk reduction. Of all statements on risk and protective factors, maintaining cognitive activity (‘stimulating your brain reduces dementia risk’) was identified most often in three samples, ranging between 88% and 91%. In the district sample including mainly people with a migration background, physical activity and depression (both 75%) were identified most often. The risk factors chronic kidney disease (between 15% and 42%) and hearing impairment (between 19% and 32%) were least identified.

**Conclusion:**

Educating the general population by raising awareness on protective and risk factors for brain health is essential. Maximizing reach and engagement of underrepresented groups is invaluable for developing interventions that help minimizing social inequalities in brain health. A dedicated awareness campaign is currently being conducted in NDPI to promote an inclusive dementia risk reduction strategy.

